# Effect of the Concrete Slurry Waste Ratio on Supercritical CO_2_ Sequestration

**DOI:** 10.3390/ma16020742

**Published:** 2023-01-12

**Authors:** Sang-Rak Sim, Dong-Woo Ryu

**Affiliations:** Department of Architectural Engineering, Daejin University, Pocheon-si 11159, Republic of Korea

**Keywords:** concrete slurry water, concrete slurry waste, ready-mixed concrete, CO_2_ sequestration, supercritical CO_2_, net-zero

## Abstract

To prevent drastic climate changes due to global warming, it is necessary to transition to a carbon-neutral society by reducing greenhouse gas emissions in all industrial sectors. This study aimed to develop carbon utilization sequestration technology that uses the concrete slurry water generated during the production of concrete as a new CO_2_ sink to reduce CO_2_ emissions from the cement industry. This was achieved by performing supercritical CO_2_ carbonation by varying the concrete slurry waste (CSW) ratio. The study’s results confirmed that, according to the CSW ratio (5 to 25%), complete carbonation occurred within only 10 min of the reaction at 40 °C and 100 bar.

## 1. Introduction

Concrete is a major structural component of buildings and one of the most extensively used core materials [1,2,3,4,5]. The demand for concrete is expected to increase further owing to the increasing demand for social infrastructure and housing, continuous urbanization, and social development in developing countries [6,7,8,9].

**Figure 1:**
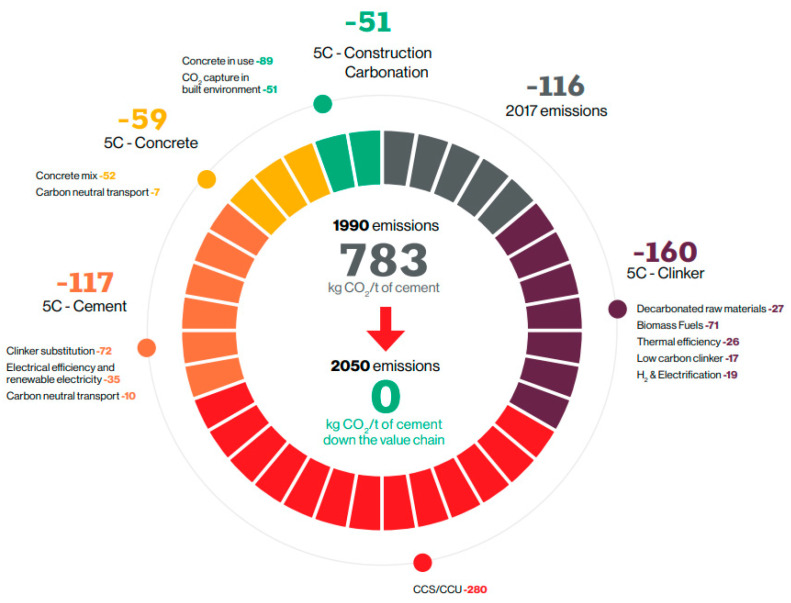
Carbon neutrality roadmap for the cement industry proposed by CEMBREAU [10].

Numerous studies have been conducted, and implementation strategies have been established worldwide to realize the 2050 carbon neutrality goals. Several initiatives have also been made in the cement industry to reduce greenhouse gas (GHG) emissions. However, global carbon emissions are increasing every year. In 10 major countries with high-GHG emissions (listed in Table 1), the carbon emissions per capita (as of 2021) are reported to exceed the global average (4.7 tons of CO_2_ per capita) [10]. In South Korea, carbon emissions are also increasing every year. At the time of the Paris climate agreement, South Korea’s GHG reduction target for 2030 was set at 37% compared with Business-as-Usual (BAU), but it was increased to 40% compared with 2018 considering the recent international trend toward GHG reduction. Accordingly, efforts have been expended in the cement industry to reduce CO_2_ emissions, including the use of blended cement and fuel conversion to increase the thermal efficiency of kilns, but they are not sufficient to meet the reduction quota. CEMBREAU presented a carbon neutrality roadmap to achieving carbon neutrality in the cement industry. According to the roadmap, various carbon emission reduction measures were prepared from clinker to concrete levels, including the increased use of blended cement and fuel conversion to improve the thermal efficiency of kilns. However, these measures alone cannot achieve carbon neutrality; thus, it is essential to develop carbon capture, utilization, and storage (CCUS) technologies, such as mineral carbonation, to achieve considerable CO_2_ reductions [11,12]. Figure 1 shows the carbon neutrality strategy of the cement industry presented by CEMBREAU.

Meanwhile, the production of ready-mixed concrete involves the generation of concrete slurry water because the returned/surplus concrete or the concrete attached to the truck agitator and batching plant mixer is washed [13,14,15]. CSW is mostly dehydrated cake produced with a filter press in a ready-mixed concrete plant after separating the fine and coarse aggregates in CSW. As CSW contains many unhydrated cement particles, studies on recycling CSW as a cementitious material are increasing every year [16,17,18,19]. Nevertheless, most of this research focuses on utilizing CSW as a filler or binder of cement matrices. However, as CSW contains a large amount of Ca^2+^ owing to unhydrated cement particles, it is expected to be highly applicable as a material for CO_2_ sequestration [20,21].

Generally, the CO_2_ mineral carbonation reaction at room temperature and atmospheric pressure is extremely slow and inefficient. Therefore, research on supercritical CO_2_ mineral carbonation has grown to accelerate the reaction. According to previous studies on mineral carbonation using supercritical CO_2_, the carbonation efficiency increases with pressure even though there are no significant differences in the influences of temperature on the carbonation reaction in the supercritical state. In most studies, the temperature and pressure are set to be less than 80 °C and 150 bar, respectively [22,23,24,25].

Therefore, in a previous study [26], mineral carbonation by supercritical CO_2_ was performed for CSW, in which the CSW ratio was adjusted to 5% at a set temperature (40 and 80 °C) and pressure (100 and 150 bars) conditions based on previous studies. It was confirmed that complete carbonation occurred within only 10 min at 40 °C and 100 bar.

**Figure 2:**
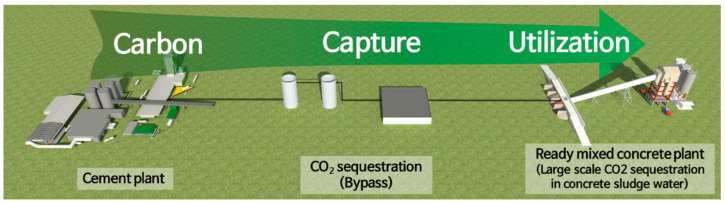
Schematic showing the sequestration of a large amount of CO_2_ in concrete slurry water.

However, it is essential to increase the proportion of CSW that contains a lot of Ca^2+^ to maximize the amount of CO_2_ fixed in concrete slurry water. According to reports, Korea produces more than 30 million tons of concrete slurry water annually [27]. Therefore, it will be possible to sequestrate a significant amount of CO_2_ generated from the cement industry if a supercritical CO_2_ carbonation process capable of continuous processing in connection with concrete slurry water in a ready-mixed concrete plant through CO_2_ capture or bypass is developed, as shown in Figure 2.

Therefore, in this study, the supercritical CO_2_ carbonation reaction was performed for 10 min at 40 °C and 100 bar with CSW, in which the CSW ratio was adjusted to its maximum of 25% as part of enforced measures to reduce GHG emissions from the cement industry. The impact of the CSW ratio on supercritical CO_2_ sequestration was also investigated using pH measurements, TG-DTA, and XRD analysis.

## 2. Experimental

### 2.1. Materials

**Figure 3:**
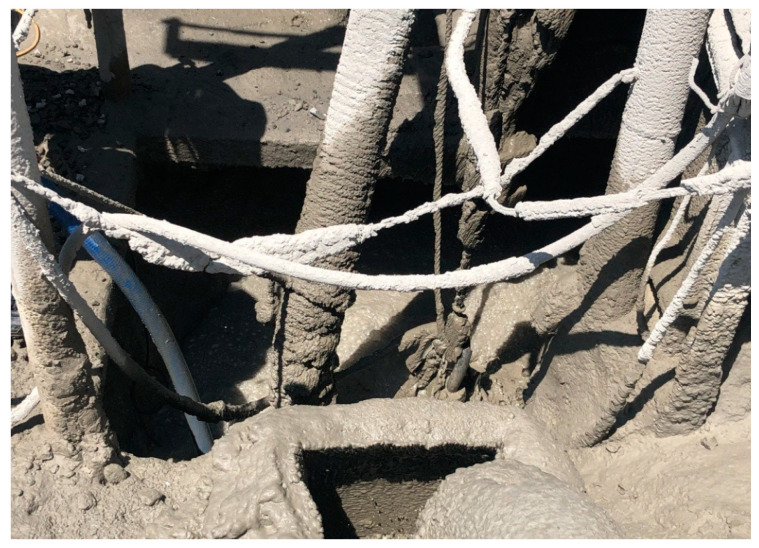
Concrete slurry water collection process.

CSW was collected from a ready-mixed concrete plant (company Y) located in Gyeonggi-do, Korea in the late afternoon, when the proportion of CSW was at its highest. Figure 3 shows the process of collecting CSW. For the CSW used in the experiment, supernatant water and CSW were separated to evenly adjust its ratio. The CSW was dried at 105 °C until a constant weight was reached. The dried CSW was then pulverized and adjusted using a No. 200 sieve (particle size ≤ 75 μm). Dilution of the supernatant water and dried CSW to the target CSW ratios (5, 10, 15, 20, and 25%) was used to perform mineral carbonation based on the supercritical CO_2_ reaction. Table 2 and Table 3 show the chemical compositions of the supernatant water and CSW, respectively. As a result of measuring the chemical composition of CSW, is was determined that it can be sufficiently used as a CO_2_ sequestration source, as it contains about 34% of the CaO component.

### 2.2. Supercritical CO_2_ Reactor

**Figure 4:**
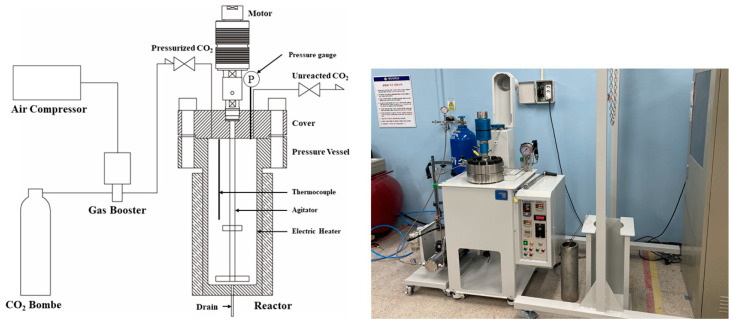
Schematic and photograph of the supercritical CO_2_ reactor.

Figure 4 shows a schematic of the supercritical CO_2_ reactor used in this study. The supercritical CO_2_ reactor consists of a gas booster (Maximator, Nordhausen, Germany) and a reactor (PHOS-ENTECH, Daejeon, Korea). A heating plate (used for temperature control) and an agitator are installed in the reactor, and a thermocouple and pressure gauge are used to measure the temperature and pressure, respectively. The gas booster is connected to the air compressor and is used to pressurize CO_2_ gas into the reactor at a high pressure to maintain the supercritical CO_2_ state. The maximum operating temperature and pressure of the supercritical CO_2_ reactor are 80 °C and 200 bar, respectively, and the internal volume of the reactor was designed to be 4 L. The agitator’s rotation speed can be adjusted to ≤400 revolutions per minute (rpm).

### 2.3. Supercritical CO_2_ Carbonation

The carbonation test was conducted using supercritical CO_2_ as follows:

The reactor was assembled after samples were added to it, in which the supernatant water and CSW had been diluted at different ratios (10, 15, 20, and 25%). When the interior of the reactor reached the target temperature of 40 °C, the electric heater was activated, and CO_2_ was injected until the target pressure of 100 bar was reached. When the CO_2_ inside the reactor reached the target pressure, the agitator was operated at 200 rpm, and accelerated carbonation was performed over a predetermined reaction time (10 min), while the temperature and pressure were maintained. After the predetermined reaction time, CO_2_ was discharged, and the reactor was disassembled to recover the sample. The supernatant water and solid content in the sample were separated, and the solid content was dried at 105 °C until it reached a constant weight. The dried solid content was subjected to pH (Hanna Instruments HI2215, Woonsocket, RI, USA), SEM (Philips XL30 ESEM, Eindhoven, The Netherlands), XRD (Rigaku D/max 2200 + Ultima, Tokyo, Japan), and TG-DTA (NETZSCH STA2500 Regulas, Germany) analysis to quantitatively evaluate the degree of carbonation reaction.

### 2.4. Measurement of Mineralogical Property Changes

The pH, SEM, XRD, and TG-DTA measurements were performed for samples before and after the carbonation reaction with supercritical CO_2_ to measure changes in the mineralogical properties of CSW.

The samples were diluted with distilled water at a 1:5 ratio before pH measurements were conducted.

TG-DTA measurements were conducted in the temperature range of up to 1000 °C at a rate of 10 °C/min in a nitrogen atmosphere to calculate the amount of CaCO_3_ generated following the reaction.

## 3. Results and Discussion

### 3.1. PH Measurements

**Figure 5:**
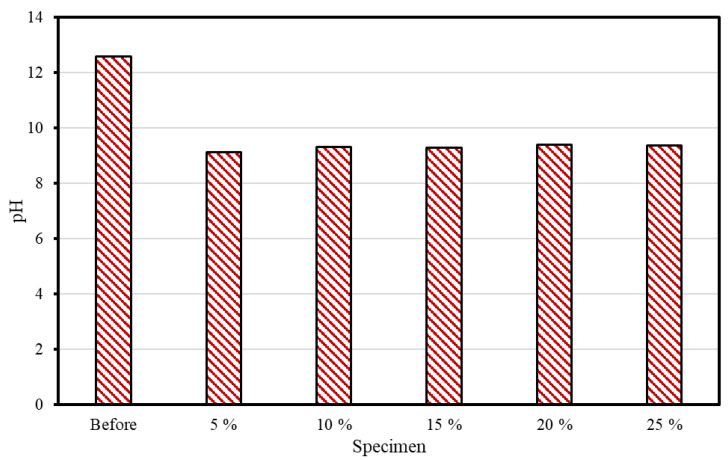
pH measurement outcomes.

Figure 5 shows the pH measurement results before and after supercritical CO_2_ carbonation. The specimen’s pH was measured to be greater than 12 before the reaction, but regardless of the CSW ratio, it ranged from 9 to 9.5 after the reaction. Typically, during the cement hydration process, Ca(OH)_2_ is produced and the pH is increased. Ca(OH)_2_ is converted into CaCO_3_ in a CO_2_-containing environment that exists during the carbonation process, as shown in Equation (1), thus resulting in a pH reduction. In this study, it appears that the carbonation reaction also caused the pH of the concrete slurry water to decrease. The pH of high-purity CaCO_3_ is known to be 9.4. It was determined that the conversion into CaCO_3_ occurred because the pH of the reaction product after supercritical CO_2_ carbonation ranged from 9.0 to 9.5.

CaO + CO_2_ → CaCO_3_ (1)

### 3.2. XRD Results

**Figure 6:**
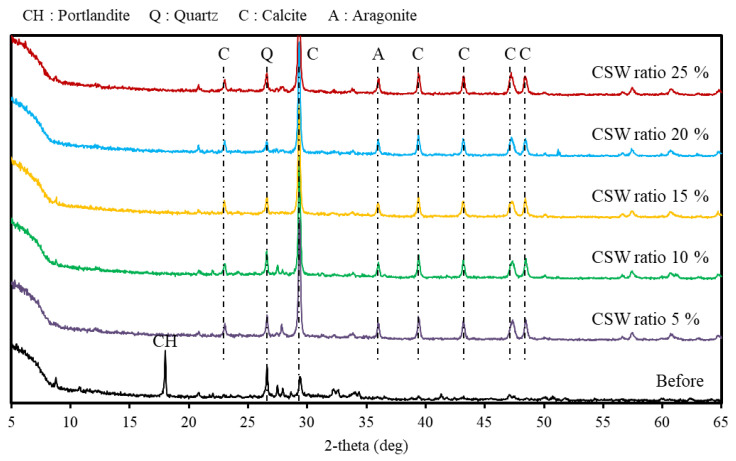
X-ray diffraction results.

Figure 6 shows the XRD measurements before and after supercritical CO_2_ carbonation. In the XRD measurement results, the Ca(OH)_2_ peak and a small amount of the calcite peak were detected from the solid sludge content before the reaction.

However, following the reaction, no Ca(OH)_2_ peak was detected, and the calcite peak was dominant in conjunction with the small aragonite peak. Additionally, similar peaks were observed regardless of the CSW ratio. CaCO_3_ is divided into aragonite, vaterite, and calcite depending on the crystal structure, and calcite is reported to be the most stable form [28]. Furthermore, a previous study reported that calcite is mainly formed when Ca^2+^/CO_3_^2−^ ≤ 1. It was determined that calcite was also mainly generated as a reaction product in this study because a significant amount of CO_3_^2−^ was generated at the supercritical CO_2_ condition that caused the Ca^2+^/CO_3_^2−^ ratio to decrease [24]. Meanwhile, quartz peaks could be confirmed both before and after the reaction. It was determined that this is due to the SiO_2_ component caused by the fine powder of the aggregate mixed in concrete slurry water [29].

### 3.3. TGA Results

**Figure 7:**
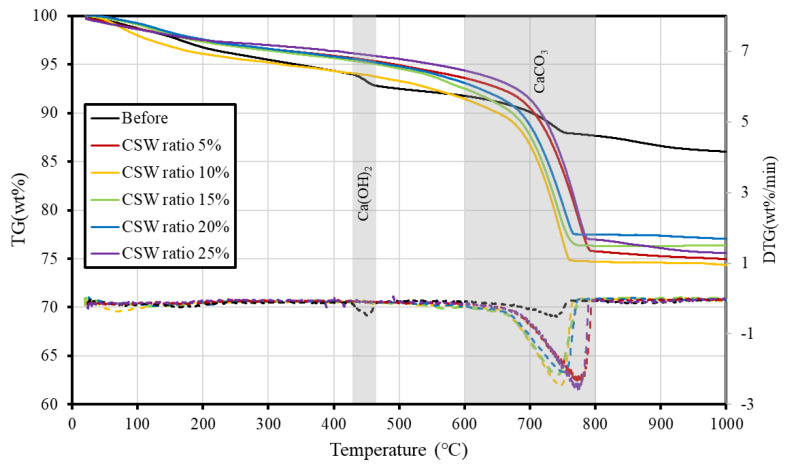
TG-DTG results (solid line: TG, dashed line: DTG).

Figure 7 shows the TG-DTA measurements before and after supercritical CO_2_ carbonation. Ca(OH)_2_, the main hydrate of cement, thermally decomposes between 400 °C and 550 °C, and CaCO_3_, which is generated following the reaction with CO_2_, thermally decomposes between 600 °C and 800 °C. In the TGA results, small weight losses of Ca(OH)_2_ and CaCO_3_ were observed in the sample before supercritical CO_2_ carbonation. However, in the samples after supercritical CO_2_ carbonation, the weight loss of Ca(OH)_2_ could not be detected, and only the weight loss of CaCO_3_ was confirmed regardless of the CSW ratio.

In the typical carbonation reaction, the formation of CaCO_3_ particles from the surface layer of particles reduces the carbonation rate in the typical carbonation reaction by inhibiting the dissolution of ions and the carbonation rate. However, according to previous studies [30,31], CaCO_3_ that has been generated on the surface of particles after carbonation can be removed through agitation; this may improve the carbonation rate by accelerating ion diffusion. Because supercritical CO_2_ is highly reactive and the agitation accelerates ionic diffusion, it is determined that complete carbonation also occurred in this study within only 10 min of carbonation reaction for CSW ratios up to 25%.

### 3.4. Evaluation of CO_2_ Sequestration

While there are many research reports on concrete carbonation using supercritical CO_2_, most studies focused on the changes in the mechanical properties and microstructure attributed to the supercritical CO_2_ carbonation of concrete [32,33,34]. Conversely, this study investigated a method for reducing industrial CO_2_ emissions and realizing net-zero emissions by developing a carbon utilization and sequestration technology that utilizes the CSW generated during concrete production as a new CO_2_ sink using the supercritical CO_2_ reaction.

Typically, it is known that when accelerated carbonation is performed on Ca(OH)_2_ or cement particles, a microcrystalline layer of CaCO_3_ is formed on the particle surfaces; as the reaction proceeds, the densification of CaCO_3_ particles in the surface layer causes the reaction to decrease gradually [22,35,36,37,38]. In this study, however, even when the CSW ratio was increased to 25%, complete carbonation occurred within only 10 min, and the reaction rate did not decrease. Previous studies [28,39] reported that when stirring is performed during the carbonation reaction, CaCO_3_ formed on the surface layer is separated from the surface of Ca(OH)_2_ particles, and the carbonation of Ca(OH)_2_ is promoted. Moreover, supercritical CO_2_ is known to be approximately 10 times more soluble in water than its dilution rate in natural carbonation conditions, and studies have reported that this can accelerate the carbonation reaction [40,41]. Accordingly, in this study, as stirring was performed during supercritical CO_2_ carbonation, CaCO_3_ particles were separated and the complete carbonation of Ca(OH)_2_ occurred despite the increased CSW ratio due to the increase in CO_2_ solubility within the CSW in supercritical CO_2_ conditions.

Meanwhile, the most common method used to evaluate the amount of sequestered CO_2_ through CCUS was the CaCO_3_ weight loss ratio achieved based on TGA analysis. Using the amount of CO_2_ produced by CaCO_3_ pyrolysis in the temperature range of 600–800 °C, the CO_2_ sequestration can be calculated by comparing the weight loss in this temperature range before and after the reaction; correspondingly, in the temperature range of 400–550 °C, the CO_2_ sequestration can be calculated by using the amount of H_2_O evaporation by Ca(OH)_2_ pyrolysis. Additionally, the Ca(OH)_2_ and CaCO_3_ contents can be calculated using Equations (2) and (3) based on the TGA results.

(2) $$Ca{\left(OH\right)}_{2}\;experiment=\left(\Delta 400\sim 550\;{}^{\circ}C\right)\times \frac{MWCa{\left(OH\right)}_{2}}{MWH_{2}O}\;$$

(3) $$CaCO_{3}\;experiment=\left(\Delta 600\sim 800\;{}^{\circ}C\right)\times \frac{MWCaCO_{3}}{MWCO_{2}}\;$$

As shown in Table 4, based on the TGA results in this study, an average weight loss of 15.67 wt% was calculated within the temperature range of 600–800 °C after supercritical CO_2_ carbonation, and the weight loss before the reaction was 2.79 wt%. This indicates that the CO_2_ sequestration ability of solid sludge is approximately 128.8 g per 1 kg. The theoretical CO_2_ sequestration based on the CaO content of solid sludge (see Equation (4)) [12] is 130.7 g per 1 kg of solid sludge. The CO_2_ sequestration calculated based on the experiment was almost identical to the theoretical at 128.8 g, thus indicating that complete carbonation occurred.

(4) $$CO_{2}\;theoretical=CaO\left(\%\right)\times \frac{MWCO_{2}}{MWCaCO_{3}}\;$$

## 4. Conclusions

The following conclusions were drawn according to the experimental results of this study.

1. When quantitative analysis (pH, XRD, and TGA) was conducted after performing the mineral carbonation of concrete slurry water by supercritical CO_2_, Ca(OH)_2_ was detected before the carbonation reaction; however, this outcome was not confirmed, and only the presence of CaCO_3_ was detected after the carbonation reaction regardless of the CSW ratio, thus confirming the occurrence of complete carbonation. It appears that complete carbonation occurred because supercritical CO_2_ is highly reactive and agitation accelerates ionic diffusion. Future studies must quantitatively analyze the CO_2_ sequestration of concrete slurry water to evaluate the possibility of reducing CO_2_ emissions from the cement industry.
2. Future research will be conducted to develop a continuous process capable of supercritical CO_2_ carbonation for CSW. By developing this technology, CO_2_ captured in cement and other industries can be transported to ready-mixed concrete plants near bases and sequestered based on rapid supercritical CO_2_ carbonation. Ultimately, the utilization of CO_2_ generated in cement and other industries will greatly contribute to the effective utilization of waste in the ready-mixed concrete industry and carbon neutrality.

## Figures and Tables

**Table 1:** Carbon emissions per capita in major countries [10].

Country	CO_2_ Emissions per Capita (tCO_2_)	CO_2_ Emissions (MtCO_2_)
China	8.0	11,472.37
United States of America	15	5007
India	1.9	2710
Russian Federation	12	1756
Japan	8.6	1067
Iran	8.5	749
Germany	8.1	675
Saudi Arabia	19	672
Indonesia	2.3	619
South Korea	12	616

**Table 2:** Chemical composition of supernatant water (obtained by ICP spectroscopy).

Chemical Composition (mg/L)				
Ca	Mg	Na	Fe	K
812	0	242	0	711

**Table 3:** Chemical composition of the concrete slurry waste (obtained by XRF spectroscopy).

Chemical Composition (wt.%)									
CaO	SiO_2_	Al_2_O_3_	Fe_2_O_3_	SO_3_	MgO	K_2_O	TiO_2_	Na_2_O_3_	P_2_O_5_
34.32	26.24	8.27	3.12	2.37	2.10	1.05	0.47	0.37	0.23

**Table 4:** Amounts of Ca(OH)_2_ and CaCO_3_ before and after supercritical CO_2_ carbonation.

	400–550 °C	Amount of Ca(OH)_2_	600–800 °C	Amount of CaCO_3_
Before	1.1	4.57	2.79	7.04
CSW 5%	0	0	15.94	40.20
CSW10%	0	0	15.84	39.94
CSW 15%	0	0	15.12	38.13
CSW 20%	0	0	15.21	38.36
CSW 25%	0	0	16.23	40.93

## Data Availability

All the data available in main text.
